# Higuchi Fractal Properties of Onset Epilepsy Electroencephalogram

**DOI:** 10.1155/2012/461426

**Published:** 2012-02-22

**Authors:** Truong Quang Dang Khoa, Vo Quang Ha, Vo Van Toi

**Affiliations:** ^1^Biomedical Engineering Department, International University of Vietnam National University, Ho Chi Minh City, Vietnam; ^2^Biomedical Engineering Department, Tufts University, MA 02155, USA

## Abstract

Epilepsy is a medical term which indicates a common neurological disorder characterized by seizures, because of abnormal neuronal activity. This leads to unconsciousness or even a convulsion. The possible etiologies should be evaluated and treated. Therefore, it is necessary to concentrate not only on finding out efficient treatment methods, but also on developing algorithm to support diagnosis. Currently, there are a number of algorithms, especially nonlinear algorithms. However, those algorithms have some difficulties one of which is the impact of noise on the results. In this paper, in addition to the use of fractal dimension as a principal tool to diagnose epilepsy, the combination between ICA algorithm and averaging filter at the preprocessing step leads to some positive results. The combination which improved the fractal algorithm become robust with noise on EEG signals. As a result, we can see clearly fractal properties in preictal and ictal period so as to epileptic diagnosis.

## 1. Introduction

Fractal dimension (FD) is considered as a important parameter applied to human biosignals. The results of FD in time domain depend on algorithm and window length. This problem was analyzed deeply by Pradhan and Dutt, when they discussed the effect of window length and window displacement on results [1].

In 2001, Echauz et al. [2] compared between results of Higuchi algorithm [3], Katz [4] and Petrosian algorithm [5] in intracranial electroencephalogram (I-EEG) epilepsy signal. The results showed that Katz's algorithm was the most consistent method for discrimination of epileptic states from the I-EEG, likely due to its exponential transformation of FD values and relative insensitivity to noise. Higuchi's method, however, yields a more accurate estimation of signal FD, when tested on synthetic data, but is more sensitive to noise. Petrosian's method performance depends on the type of binary sequence used. If a binary sequence based on slope-sign changes is utilized then this method becomes less suitable for analog signal analysis, given its high sensitivity to noise and its poor reproducibility of dynamic range of synthetic FDs. Kannathal et al. [6] used Katz algorithm and Higuchi algorithm to calculate averaging fractal dimensions of 2 groups: one is healthy, another is epilepsy patient. Results show that the FDs of the epilepsy group are lower than healthy one in both methods. In epilepsy detection, Esteller et al. [7] said that by the time seizures happened, the fractal dimension using Katz algorithm increases in the ictal period, followed by a fall to the lowest complexity level of the recording. Moreover, in 2003, this group used 6 parameters, including curve length, energy, nonlinear energy, spectral entropy, sixth power, and energy of wavelets packets, as features for EEG segmentation in epilepsy [8]. In the same way, Bao et al. used Higuchi Algorithm, Petrosian algorithm, Hjorth parameters, power spectra, means, standard deviation, and neural network for epilepsy diagnosis [9].

In this study, we analyze the fractal properties as parameters for both EEG and ECG epilepsy detection.

## 2. Methology

**Figure 1:**
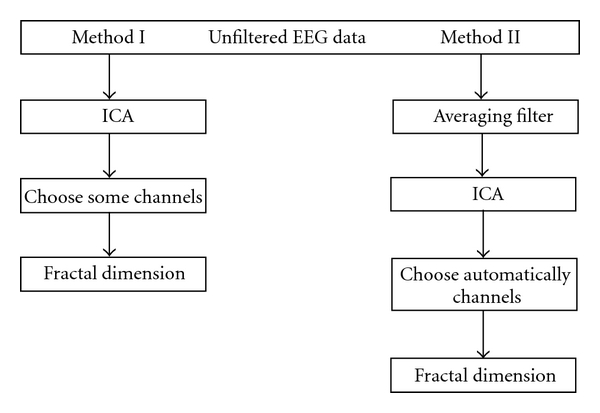
Proposed algorithms.

In this paper, we proposed two methods to analyze epilepsy data. The first method includes two steps: all of channels were analyzed to archive independent components by ICA algorithm. After that, Higuchi algorithm was used to calculate fractal dimension. The second method processed the same way to the first method, except that an averaging filter was used as the first. The methodology used in this paper consists of the steps shown in the diagram in Figure 1.

### 2.1. Averaging Filter (AF)

The averaging filter is the simplest type of low-pass filter using when the neighborhood considered is too large blurring and other unwanted effects can appear in the data set. This method can be useful to avoid very high frequency noise and white noise. The value of a sample is calculated by the average of its neighbors:

(1) $$x_{n}=\frac{1}{2k+1}\overset{k}{\underset{i=-k}{\sum }}x_{n+i},$$

where *k* is the window length, *x*_*n*_ is the value of *n*th sample.

 By experiments, we assume that *k* = 3 is suitable for epilepsy prediction.

### 2.2. Independent Component Analysis (ICA)

#### 2.2.1. Definition of ICA

We assume that we observe *n* linear mixtures *x*_1_,…, *x*_*n*_ of n independent components:

(2) $$x_{j}=a_{j1}s_{1}+a_{j2}+\cdots +a_{jn}s_{n},\;j=\bar{1,n}.$$

We have now dropped the time index *t*; in the ICA model, we assume that each mixture *x*_*j*_ as well as each independent component *s*_*k*_ is a random variable, instead of a proper time signal [10]. Without loss of generality, we can assume that both the mixture variables and the independent components have zero mean; if this is not true, then the observable variables *x*_*i*_ can always be centered by subtracting the sample mean, which makes the model zero mean:

(3) $$\tilde{x}=x-E\left(x\right).$$

Let *x* be the random vectors whose elements are the mixtures *x*_1_,…, *x*_*n*_ and let s be the random vector with the components *s*_1_,…, *s*_*n*_. Let *A* be the matrix containing the elements *a*_*ij*_. The model can now be written as follows:

(4) $$x=As\;\text{Or}\;x=\overset{n}{\underset{i=1}{\sum }}a_{i}s_{i}.$$

The above equation is called independent component analysis or ICA. The problem is to determine both the matrix *A* and the independent components *s*, knowing only the measured variables *x*. The only assumption the methods take is that the components *s*_*i*_ are independent. It has also been It has also been proved that the components must have nongaussian distribution.

Before the application of the ICA algorithm (and after centering), we transform the observed vector *x* linearly to obtain a new vector $\tilde{x}$ which is white (its components are uncorrelated and their variances equal unity).

Whitening can be performed via eigenvalue decomposition of the covariance matrix:

(5) $$E\left\{xx^{T}\right\}=EDE^{T},$$

where *E* is the orthogonal matrix of eigenvectors of *E*{*xx* ^*T*^} and *D* is the diagonal matrix of its eigenvalues, *D* = diag⁡(*d*_1_,…, *d*_*n*_). Whitening can now be done by

(6) $$\tilde{x\;}=ED^{-1/2}E^{T}x.$$

#### 2.2.2. Fast ICA for *n* Units [10]

A unit represents a processing element, for example, an artificial neuron with its weights *W*.

To estimate several independent components, the weights *w*_1_,…, *w*_*n*_ must be determined. The problem is that the outputs *w*_1_^*T*^ *x*,…, *w*_*n*_^*T*^ *x* must be done as independent as possible after each iteration in order to avoid the convergence to the same maxima. One method is to estimate the independent components one by one.

**Algorithm 2.2.2**

**Step 1**

Initialize *w*_*i*_.

**Step 2**

Newton phase:

(7) $$w_{i\;}=\;E\left\{\tilde{x}g\left(w_{i\;}^{T}\tilde{x}\right)\right\}-\;E\left\{g^{\prime }\left(w_{i}^{T}\tilde{x}\right)\right\}w_{i},$$

where *g* is a function with one of the following forms:

(8) $$\begin{array}{c} g_{1}\left(y\right)=\tanh\left(a_{1}y\right),\\ g_{2}\left(y\right)=y\exp\left(-\frac{1}{2}y^{2}\right),\;\;g_{3\;}\left(y\right)=4y^{3}. \end{array}$$

**Step 3**

Normalization:

(9) $$w_{i}=\frac{1}{||w_{i}||}w_{i}.$$

**Step 4**

Decorrelation:

(10) $$w_{i}=w_{i}-\overset{i-1}{\underset{j=1}{\sum }}w_{i}^{T}w_{j}w_{i}.$$

**Step 5**

Normalization (like in the Step 3).

**Step 6**

Go to Step 2 if not converged.

#### 2.2.3. Higuchi's Fractal Dimension Algorithm

Higuchi's algorithm calculates fractal dimension of a time series directly in the time domain. It is based on a measure of length, *L*(*k*), of the curve that represents the considered time series while using a segment of *k* samples as a unit, if *L*(*k*) scales like

(11) $$L\left(k\right)\sim k^{-D_{f}}.$$

The curve is said to show fractal dimension *D*_*f*_ because a simple curve has dimension equal 1 and a plane has dimension equal 2; value of *D*_*f*_ is always between 1 (for a simple curve) and 2 (for a curve which nearly fills out the whole plane). *D*_*f*_ measures complexity of the curve and so of the time series this curve represents on a graph.

From a given time series, *X*(1), *X*(2),…, *X*(*N*), the algorithm constructs *k* new time series:

(12) $$\begin{array}{c} X_{km}:X\left(m\right),X\left(m+k\right),X\left(m+2k\right),\ldots ,\\ X\left(m+\mathrm{int}\left(\frac{\left(N-m\right)}{k}\right)\cdot k\right)\;\text{for}\;m=1,2,\ldots ,k, \end{array}$$

where *m* is initial time, *k* is interval time, int(*r*) is integer part of a real number *r*.

 For example, for *k* = 4 and *N* = 1000, the algorithm produces 4 time series:

(13) $$\begin{array}{cc} X_{41} & :X\left(1\right),X\left(5\right),X\left(9\right),\ldots ,X\left(997\right),\\ X_{42} & :X\left(2\right),X\left(6\right),X\left(10\right),\ldots ,X\left(998\right),\\ X_{43} & :X\left(3\right),X\left(7\right),X\left(11\right),\ldots ,X\left(999\right),\\ X_{44} & :X\left(4\right),X\left(8\right),X\left(12\right),\ldots ,X\left(1000\right), \end{array}$$

The “length” *L*_*m*_(*k*) of each curve *Xkm* is then calculated as

(14) $$\begin{array}{cc} L_{m} & =\frac{1}{k}\left[\left(\overset{\mathrm{int}(\left(N-m\right)/k)}{\underset{i=1}{\sum }}\left|X\left(m+i\cdot k\right)-X\left(m+\left(i-1\right)\cdot k\right)\right|\right)\right]\\ & \;\times \frac{N-1}{\mathrm{int}\left(\frac{\left(N-m\right)}{k}\right)\cdot k}, \end{array}$$

where *N* is total number of samples.

*L*_*m*_(*k*) is not “length” in Euclidean sense, it represents the normalized sum of absolute values of difference in ordinates of pair of points distant *k* (with initial point *m*). The “length” of curve for the time interval *k*, *L*(*k*), is calculated as the mean of the *k* values *L*_*m*_(*k*) for *m* = 1,2,…, *k*:

(15) $$L\left(k\right)=\frac{\sum_{m=1}^{k}L_{m}\left(k\right)}{k}.$$

The value of fractal dimension, *D*_*f*_, is calculated by a least-squares linear best-fitting procedure as the angular coefficient of the linear regression of the log-log graph of (1):

(16) y=ax+b

with *a* = *D*_*f*_, according to the following formulae:

(17) $$Df=\frac{n\sum \left(x_{k}\cdot y_{k}\right)-\sum x_{k}\sum y_{k}}{n\sum x_{k}^{2}-{\left(\sum x_{k}\right)}^{2}},$$

where *y*_*k*_ = ln⁡*L*(*k*), *x*(*k*) = ln⁡(1/*k*). *k* = *k*_1_,…, *k*_max⁡_, and *n* denotes the number of *k* values for which the linear regression is calculated (2 ≤ *n* ≤ *k*_max⁡_).

The standard deviation of *D*_*f*_ is calculated as

(18) $$S_{Df}=\sqrt{\frac{n\cdot \left[\sum y_{k}^{2}-Df\cdot \sum x_{k}y_{k}-b\cdot \sum y_{k}\right]}{\left(n-2\right)\cdot \left[n\cdot {\sum x_{k}}^{2}-{\left(\sum x_{k}\right)}^{2}\right]}},$$

where

(19) $$b=\frac{1}{n}\left(\sum y_{k}-Df\cdot \sum x_{k}\right),$$

with standard deviation

(20) $$S_{b}=\sqrt{\frac{1}{n}\cdot S_{Df}^{2}\cdot \sum x_{k}^{2}}.$$

Higuchi's fractal dimension has a scaling feature. Multiplication of all amplitudes *X*_*km*_ by a constant factor, *c*, causes multiplication of the “length” *L*_*m*_(*k*) by the same factor. Such multiplication does not change *D*_*f*_:

(21) $$Ln\left(L\left(k\right)\right)=Df\cdot \ln\left(\frac{1}{k}\right)+\left(b+\ln\left(c\right)\right).$$

Window length has a meaning effect to the results. Because seizures spread so quickly, a displacement as small as possible that does not provide too much variability is desired. We experimented with values ranging from 1 second to 60 seconds and observed that the window length to 2048 points (16 seconds) with 50% overlap should provide reasonable propagation resolution of seizure precursors and the ability of multichannel analysis to effect detection.

## 3. Results and Discussion

**Figure 2:**
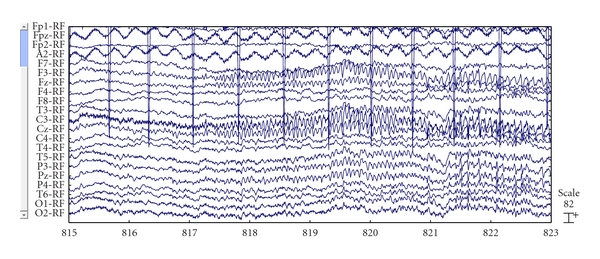
The recording of an epilepsy patient.

Figure 2 shows an EEG recording of an epilepsy patient which lasted in the vicinity of 21 minutes.

According to the record, it was different between before and after 848th second (14 minutes 08 seconds). Before this point of time, data showed that the neuronal activities were chaotic. However, after that, the brain activity was periodic as a series of high-frequency repetitive spikes. Therefore, it has the ability on seizure onset detection which can probably rely on alteration in fractal characteristic of the signal calculated by Higuchi algorithm. It should be noted that because Higuchi algorithm is so sensitive to noise, preprocessing step should be concentrated on to obtain the most believable results. Therefore, in this study, the preprocessing procedure was carried on by two methods which are described below.

### 3.1. Method 1

**Figure 3:**
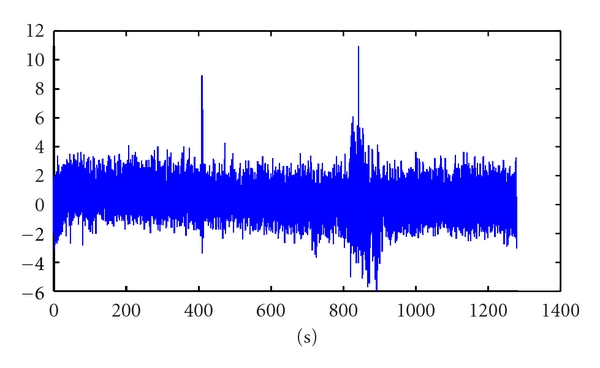
The main component containing epilepsy wave following Method 1.

After being analyzed by ICA algorithm, the main component which contains epilepsy wave was illustrated on Figure 3.

**Figure 4:**
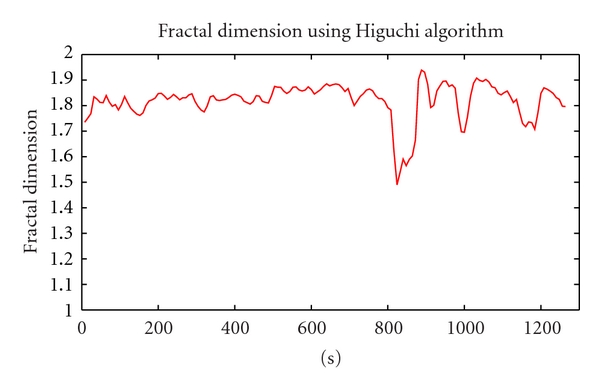
Fractal Dimension of IC22 channel.

As can be seen in Figure 3, there were 2 periods of time which had a considerable fluctuation with high amplitude than others. While the first was caused by stimulation effect, the second was the ictal period. The result of FD using Higuchi algorithm is shown in Figure 4.

As regard to Figure 4, the most remarkable aspects of these trends are, during the preictal period, the fractal dimension was relatively high and erratic fluctuates in a small range, hovering at 1.7. This pattern lasted about 13 minutes, until the fractal dimension number reached a peak at 736th second (the window length is 16, overlap 50%). Then the graph declined gradually to 800th second, followed by a sharp fall from 818th second to the trough at 848th seconds. The figure then experienced a recovery, reached to the maximum before falling down to the initial state. The most prominent meaning is that the beginner of ictal period in original data corresponds to the minimal drop in the FD values. Before minimum point occured appoximately in 2 minutes, the fractal dimension value started to decline. Therefore, it is possible to predict some minutes before the happening of seizure.

However, because Higuchi algorithm is very sensitive to noise [2], especially white noise, the average Fractal dimension of each channel in data is so high and it is so difficult to detect epilepsy. The current difficulty is that we cannot know exactly where the main component from results of ICA is processing. Therefore, we propose using averaging filter for the original data. The results of this method will be described clearly below.

### 3.2. Method 2

**Figure 5:**
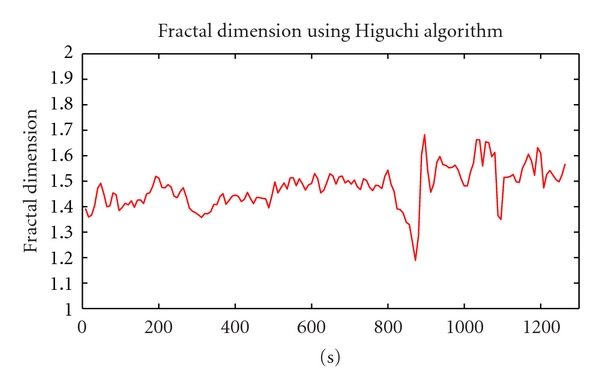
The main component containing epilepsy wave following Method 2.

According to the Figure 1, the original data experienced two filtering stages before calculating fractal dimension of obtained components. Based on the value of fractal dimension, the results can be separated into two groups of ICA components. Components which had high average fractal dimension value had the same patterns with method 1: during the preictal period the fractal dimension was relatively high and remained stable, the fractal dimension exhibited an substantial decrease during the initial stage of the ictal period, and then it went up again, reaching to a peak, followed by a fall to normal state. Meanwhile, the sign of epilepsy did not appear in the balance group. Therefore, the component which had the highest fractal dimension can be considered as the main components that were showed in Figure 5.

The combination between average filter and ICA brings to us quality results. The main reason is that the advantage of averaging filter can probably reject high frequency components of external noisy source which affected mainly on the result of Higuchi algorithm, while ICA is good at rejecting internal noise. From this combination, we can obtain the main component which contained epilepsy waves.

In averaging filter formulae, the length of the window, *k*, should not be selected too large to lose information of epilepsy wave. This step is suitable for rejecting random noise or very high frequency noise. Therefore, this is an appropriate method in Vietnam condition where equipment, faculty, and measurement condition are not very good. There are not many hospitals applying Faraday cage which is used to eliminate effects of noisy environment.

We noticed that the fractal dimension calculated by Higuchi algorithm has a high degree of accuracy [2]. But, it is very sensitive to noise. So, the step of noise rejection is really important in this research. Using ICA to keep signal separate from noise is not a new way, however, it is so useful in this research. The difficulty when we use this algorithm is that its results include “blind channels”. Therefore, we cannot identify where sources of seizure onset are and which channels have epilepsy wave. The method 2 only helps us to choose which channels to analyze in next step. This is advantage of this method.

The trend of fractal dimension in ictal period has a slight difference from the results of Esteller et al. [7]. Their results showed that the fractal dimension in ictal period is higher than that in the preictal period and ends with a drop to the lowest complexity while the trend of our results obtained an opposite pattern. However, the alteration pattern of the complexity in this study is similar to the results of Iasemidis et al. [11] when they used the Lyapunov exponent for epilepsy data.

### 3.3. Detect Epilepsy on ECG

Besides achievements in EEG, fractal properties of ECG are also useful for epilepsy diagnosis. While epileptic sign can be visually observed on EEG records, ECG is not paid attention to be considered as a mean playing a substantial role in diagnosing epilepsy. However, in this study, we also attempted to estimate fractal characteristics of ECG of epileptic patients.

**Figure 6:**
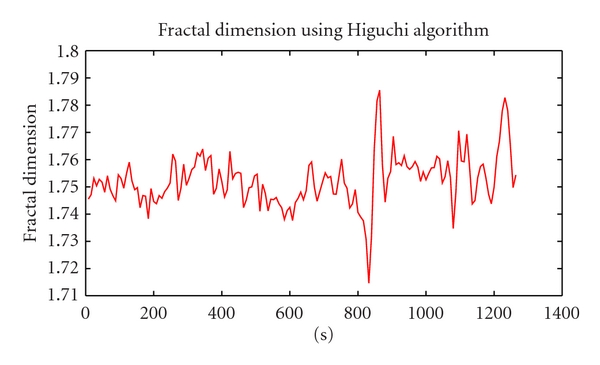
The result of fractal dimension on ECG channel.

According to Figure 6 obtained by the Higuchi algorithm, we can see that fractal coefficient of ECG turned for the worse in the transition from preictal to ictal period. That general pattern was very close to the result of EEG when the fall of fractal figure was marked as the beginning of the seizure. In addition, before the seizure by several minutes, there were two troughs that need to be focus on in anticipation of the seizure. That issue had been discussed in study of Iasemidis et al. [11]. That fact makes a proposal that ECG is likely to become a potential method for diagnosis in that domain.

In reality, there is a variety of conveniences of processing ECG in comparison with EEG. Firstly, the former is less sensitive to noise with the great preference for the latter, the main reason is that the amplitude of ECG obtained by the sensors is far higher than that of EEG signal. Secondly, ECG is more widely used than EEG and more suitable for long-term or even perpetual inspection. Therefore, that issue needs to be discussed more deeply because of its advantages.

## 4. Conclusions

Noise is a serious problem with EEG signal processing, especially in Higuchi algorithm. Therefore, this study concentrated on developing a robust algorithm in the preprocessing step which was the combination between ICA and averaging filter. This fact aimed to reject some kinds of internal and external noise. In addition, this study shows the fractal dimension properties in EEG of epilepsy patients. The results also suggest that FD is a practical tool for identification of seizure onset in the EEG data. The changes in EEG from unperiodic to periodic signal show clearly through the alterations of fractal coefficient to the minimal point. These FD changes may provide insight into the underlying dynamics of this unknown system. These methods can open the possibility of designing an intelligent system for predicting and warning of seizures in real time as a preference or a standard of expert visual analysis of electrographic seizure onset. Moreover, the existing of epileptic sign in fractal result of ECG should be paid attention because of the advantages that could bring to us.
